# Atypical Presentation of Idiopathic Retroperitoneal Fibrosis Effectively Treated With Colchicine After Lymphoma Misdiagnosis

**DOI:** 10.7759/cureus.14756

**Published:** 2021-04-29

**Authors:** Ellery Altshuler

**Affiliations:** 1 Internal Medicine, University of Florida College of Medicine, Gainesville, USA

**Keywords:** idiopathic retroperitoneal fibrosis, colchicine, abdominal aorta, igg4, autoimmune, lymphoma, acute pulmonary embolism

## Abstract

Idiopathic retroperitoneal fibrosis (RPF) is a rare disease characterized by a fibro-inflammatory mass encasing the abdominal aorta. We report a case of a 43-year-old man with an unusual presentation of RPF who was initially misdiagnosed with lymphoma. Our patient presented with constipation and did not have common findings such as ureteral displacement or renal impairment. Our patient had a complicated disease course complicated by multiple treatment failures and pulmonary embolism. We discuss the patient's first 100 months of treatment, which included the use of prednisone, mycophenolate, tamoxifen, methotrexate, azathioprine, and, now, colchicine. Our case demonstrates that physicians should maintain an index of suspicion for RPF in patients with a homogenously attenuated mass encasing the anterior aorta. It also serves as one example in which RPF appeared to be responsive to colchicine.

## Introduction

Idiopathic retroperitoneal fibrosis (RPF) is characterized by a fibro-inflammatory mass encasing abdominal aorta [1]. Pathogenesis is thought to be related to an exaggerated immune reaction [2]. RPF can be a solitary disease or part of a systemic autoimmune disorder [2]. When associated with systemic autoimmune disease, it is considered to be on the spectrum of IgG4-related fibro-inflammatory disorders, which involve irregular fibrosis associated with IgG-4-positive plasma cells [3].

## Case presentation

**Figure 1:**
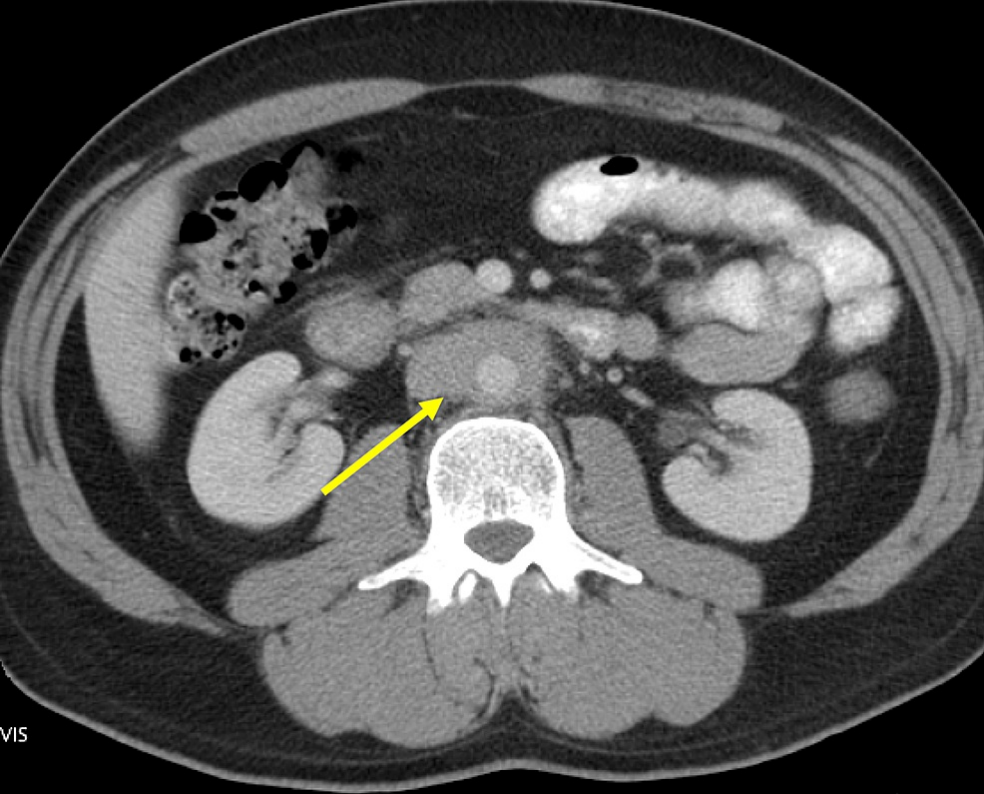
CT that was used to diagnose lymphoma. Visible is a periaortic infiltrating mass (arrow) and mildly reactive lymph nodes. Note that lymph nodes are non-confluent, the aorta is not displaced anteriorly, the bones are intact, and there is no psoas muscle dislocation.

A 43-year-old man with a past medical history significant only for migraines presented with a three-month history of abdominal pain and constipation. The pain was worse with eating and the patient had lost 35 pounds. He endorsed constipation and ribbon-like stools. He had several episodes of bilious vomiting and endorsed tingling in the lower extremities but denied dysuria. Of note, the patient reported taking Cafergot (ergotamine tartrate and caffeine) for migraines for several months about a decade prior to presentation. He was a former smoker with 25 pack-years who had quit 10 years prior to admission. CT imaging revealed a periaortic infiltrating mass involving the infrarenal abdominal aorta with mildly prominent lymph nodes thought to represent a malignancy (Figure 1). The mass was not amenable to percutaneous biopsy. The patient was told he had lymphoma and referred to oncology.

**Figure 2:**
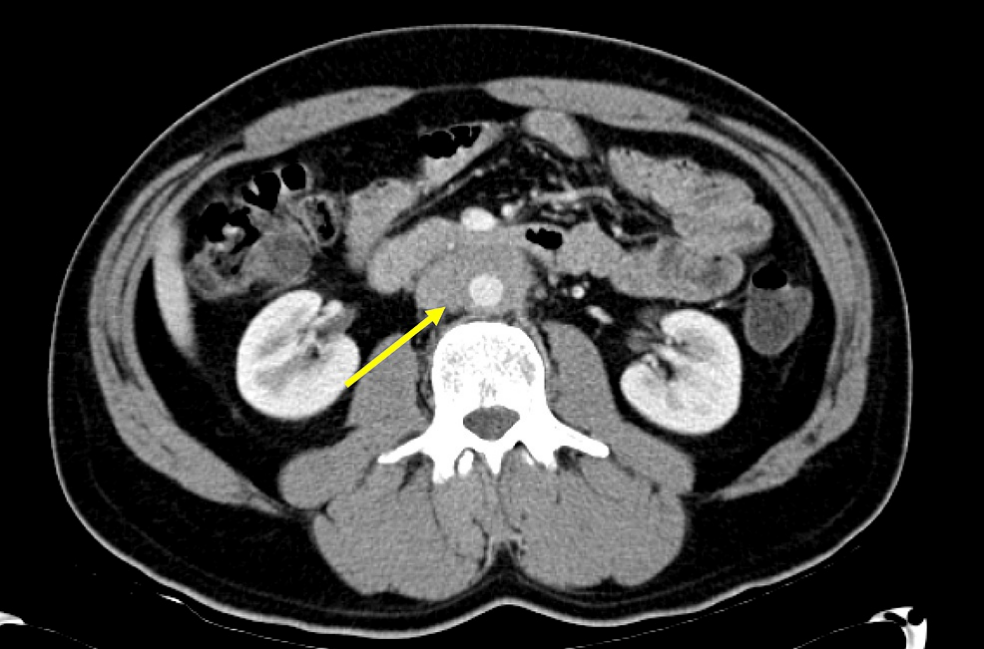
Repeat CT at the time of diagnosis. Circumferential soft tissue thickening around the distal abdominal aorta and proximal right common iliac measuring up to 1 cm in thickness (arrow). There is no appreciable hydronephrosis or ureteral displacement.

The diagnosis of lymphoma was doubted by the oncologist. On physical exam, there was no palpable lymphadenopathy or hepatosplenomegaly. Repeat CT scan again revealed circumferential soft tissue thickening around the distal abdominal aorta and proximal right common iliac artery but, this time, the radiologist recognized that the findings were more consistent with retroperitoneal fibrosis (Figure 2).

**Figure 3:**
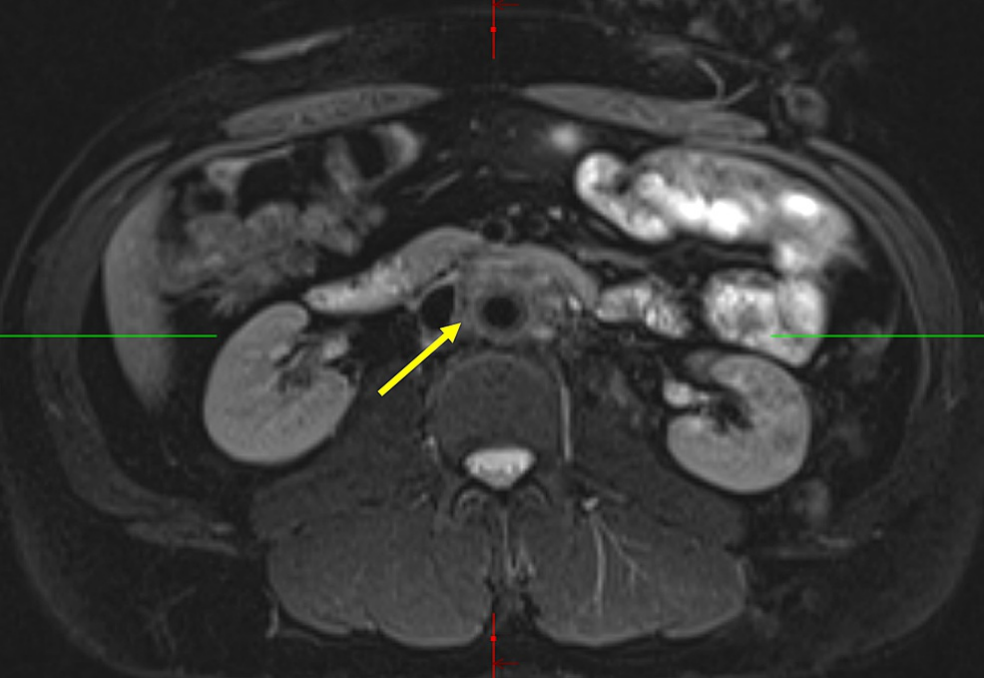
MRI at the time of diagnosis Circumferential soft tissue thickening (arrow) is clearly visible around the infrarenal aorta. The ureters are not retracted into the soft tissue thickening and there is no hydronephrosis.

MRI imaging also demonstrated soft tissue thickening around the infrarenal aorta and showed mild T2 hyperenhancement of the mass (Figure 3).

The patient's labs were mostly normal (Table 1). No electrolyte abnormalities were noted and urinalysis was unremarkable. Blood urea nitrogen (BUN) and creatinine were normal (and have remained within normal limits over the last eight years). Tests for autoimmune diseases -- including antinuclear antibody (ANA), rheumatoid factor, and cyclic citrullinated peptide (CCP) -- were all negative. Infectious disease workup was revealing only for cytomegalovirus (CMV) antibodies and negative for hepatitis B, hepatitis C, and TB. EGD and colonoscopy done to evaluate for concomitant gastrointestinal pathologies were normal. IgG4 levels were normal at 20.3 mg/dL (reference range: 4.0-86.0 mg/dL). They have been rechecked several times since then without significant change.

**Table 1:** Selected lab values at the time of diagnosis WBC: white blood cells; Hgb: hemoglobin; HCT: hematocrit; BUN: blood urea nitrogen; EGFR: estimated glomerular filtration rate; CRP: c-reactive protein; CEA: Carcinoembryonic antigen.

Test	Value	Reference Range
WBC	8.1 thousand/uL	3.8-10.8 thousand/uL
Hgb	12.4 g/dL	13.5-17.5 g/dL
HCT	37%	41%-53%
Platelet Count	329 thousand/uL	140-400 thousand/uL
BUN	9 mg/dL	7-25 mg/dL
Creatinine	0.93 mg/dL	0.84-1.21 mg/dL
EGFR	100 mL/min/1.73m^2^	60 mL/min/1.73m^2^
CRP	0.3 mg/L	<1.0 mg/L
CEA	<0.5 ng/mL	<2.5 ng/mL

**Figure 4:**
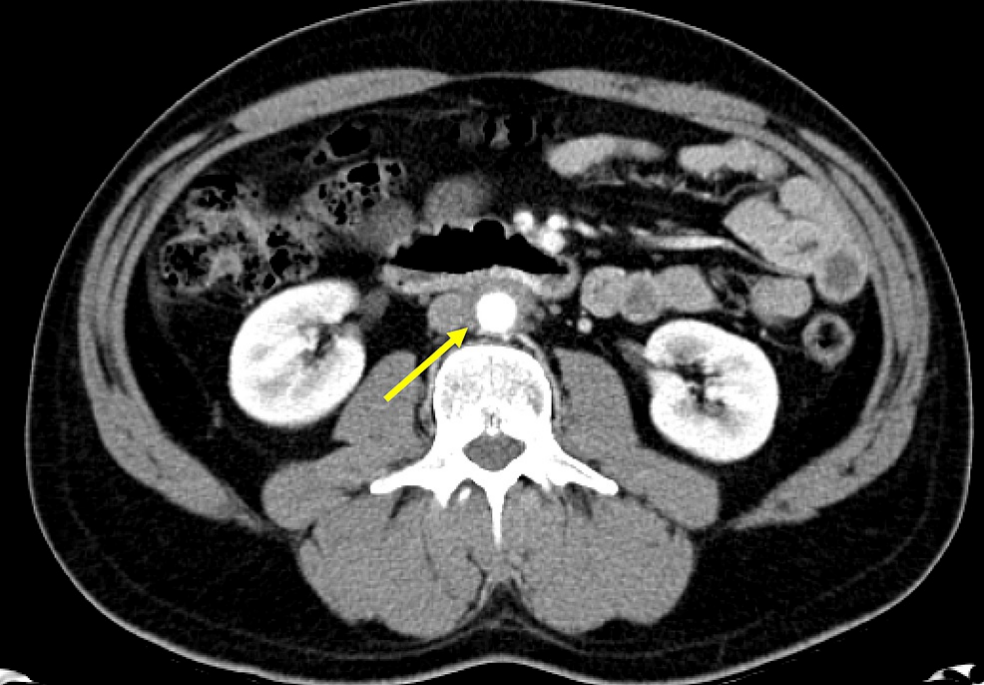
CT three months after initiation of treatment with prednisone demonstrating treatment effect There is a marked improvement in the circumferential soft tissue thickening (arrow) around the infrarenal abdominal aorta. This circumferential soft tissue measures up to 5 mm in thickness (decreased from 1 cm on prior CT). Again, there is no ureteral retraction or hydronephrosis.

He has received several different treatments for RPF. He was initially treated with a month of daily prednisone 80 mg soon after his diagnosis and showed significant improvement in his symptoms and a marked decrease in the size of his mass on CT (Figure 4). He was maintained on low-dose prednisone for nine months. Tamoxifen was trialed during this time but was not tolerated due to sexual side effects.

He was then switched to mycophenolate and, after six months of minimal symptoms, all medications were stopped (Table 2). Symptoms returned, however, and a rescue steroid taper was required. Methotrexate was used for 18 months with good response, but side effects became difficult to tolerate. Azathioprine was then used for 11 months and also appeared to lessen disease burden but was not well tolerated. Colchicine was started and constipation and abdominal pain have been well-controlled since then. His imaging findings have remained unchanged and he feels generally well.

**Table 2:** Medications used over first 100 months of treatment

Time from diagnosis	Medication	Symptom Severity	Imaging	Reason regimen changed
0-3 months	Prednisone 80 mg, followed by taper	Mild	CT scan shows 50% reduction in retroperitoneal fibrosis (Figure 4).	Completion of course of steroids
3-12 months	Prednisone, 10 mg daily Tamoxifen not tolerated.	Mild	Stable to minimally improved	Worsening of disease
12-18 months	Mycophenolate 1000 mg twice daily.	Mild	Stable disease	Patient desire to be off medication given stability of disease, mild side-effects
18-36 months	None	Severe	Clear evidence of worsening disease with new-onset mild right-sided hydronephrosis	Worsening of disease
36-39 months	Prednisone 80 mg daily, followed by taper	Mild	Substantial improvement, no evidence of hydronephrosis	Completion of course of steroids
39-57 months	Methotrexate, 20 mg weekly prednisone 5 mg daily	Mild	Stable	Fatigue, weakness, and pain attributed to methotrexate
57-63 months	None	Severe	Worsened tissue thickening surrounding aorta	Worsening of disease
63-74 months	Azathioprine, 150 mg daily	Mild	Stable	Nausea/vomiting attributed to azathioprine
74-100 months	Colchicine 1.2 mg daily	Mild	Stable	Ongoing

The patient's clinical course was complicated by a pulmonary embolism at 100 months from diagnosis involving bilateral lobar and segmental branches. He had no preceding immobilization, illness, surgery, or trauma prior to the event. He was hospitalized with acute hypoxic respiratory failure that resolved with heparin anticoagulation. He was discharged in stable condition on apixaban.

## Discussion

RPF is rare, with an incidence is about 1-3 new cases per million people per year and a prevalence of about 14 cases per million people [4]. The pathogenesis of the disease is poorly understood and more than 75% of cases are considered idiopathic; the disease is associated with radiotherapy, certain drugs such as beta-agonists and ergot alkaloids, asbestos, and cigarette smoking [4,5]. Our patient's primary risk factors were cigarette smoking and the use of an ergot drug. In one case control, people with RPF were 10 times as likely as those without to have taken ergot-derived medication did (OR 1.63-60.26) [6]. He was also at higher risk due to his sex; males are affected two to three times as frequently as females [7]. The median age of onset is 55-60 and mortality at five years from diagnosis is 3%-7% [1,7].

Retroperitoneal fibrosis is classically associated with obstructive uropathy with subsequent renal impairment, and most patients with RPF present with elevations in BUN and creatinine (ESR and CRP are also used as clinical markers of disease response) [1,4]. As our case demonstrates, however, the disease should not be excluded because of normal markers of renal function. Clinical symptoms vary but usually involve back, flank, or abdominal pain [4]. In three clinical series involving a combined 286 patients, constipation -- the most persistent symptom in our patient -- was reported in fewer than a fifth of cases [4,7,8].

Our patient was initially diagnosed with lymphoma, thus underlying the importance of recognizing radiographical features that characterize RPF. On CT scan, RPF appears as a homogenously attenuated mass surrounding the anterior and lateral sides of the aorta [9]. Lymphoma, on the other hand, is not homogenous and presents with confluent lymphadenopathy [4]. On T1 MRI images, RPF appears as a hypointense, homogenous mass (intensity in T2 images varies depending on the stage of the disease and extent of tissue edema) [10]. Bone destruction and psoas muscle dislocation are associated with malignancy but not RPF [11]. Lymphadenopathy may be present in RPF -- as was the case in our patient -- but the reactive lymph nodes are localized, non-confluent, and usually measure less than a centimeter [4]. RPF frequently causes medial displacement of the ureters and rarely causes anterior displacement of the aorta; lymphoma, meanwhile, often causes lateral displacement of the ureters and commonly causes anterior displacement of the aorta [11]. As demonstrated by our patient, who did not have ureteral displacement, however, ureteral displacement is not necessary for the diagnosis of RPF. Other retroperitoneal pathologies are easier to distinguish from RPF [11]. These include inflammatory pseudotumor, desmoid tumors, and infectious etiologies such as tuberculosis and actinomycosis [11]. Tissue biopsy is usually unnecessary for diagnosis, which can be made with CT or MRI imaging.

First-line therapy is high-dose glucocorticoids, which are associated with a mean mass reduction of about 50% and the achievement of clinical remission in up to 95% of cases [4]. Without maintenance therapy, relapse may occur [4]. Common maintenance treatments involve immunosuppression with methotrexate, azathioprine, mycophenolate, and cyclophosphamide [4]. Tocilizumab and rituximab have traditionally been used as second-line agents [4].

Our patient failed multiple agents and experienced disease progression whenever he stopped taking maintenance medication. He eventually found sustained relief with colchicine, which he tolerated well. No randomized control trial exists to support the use of colchicine in patients with RPF [12]. In one study -- a case series involving six patients treated with colchicine as the initial choice of therapy after steroid taper -- four patients achieved symptomatic improvement with colchicine alone over a period of six years [12]. To the best of our knowledge, our patient represents the fifth reported case of a patient with RPF to achieve sustained response with the drug. Colchicine -- which commonly causes diarrhea -- may be a good choice in patients like ours in which the primary manifestation of RPF is constipation.

Deep vein thrombosis and venous thromboembolism are uncommon in patients with RPF [13]. Although fibrosis can impair retroperitoneal venous return and thus cause lower extremity venous stasis, this process occurs so slowly that collateral circulation can develop [13]. Due to the low prevalence of pulmonary emboli in patients with RPF, we decided to treat our patient's pulmonary embolus as an unprovoked event. Accordingly, we plan to keep the patient on therapeutic anticoagulation indefinitely.

## Conclusions

Physicians should maintain an index of suspicion for RPF in patients with a retroperitoneal mass even in the absence of renal impairment. Radiographic features distinguish RPF from lymphoma and other retroperitoneal masses. RPF appears as a homogenously attenuated mass surrounding the anterior and lateral sides of the aorta. The fibrosis is hypointense on T1 images, rarely causes displacement of the aorta, and frequently -- but not always -- causes medial ureteral displacement. Lymphoma, by contrast, often causes lateral displacement of the ureters and anterior displacement of the aorta. No randomized control trials exist for the long-term treatment of persistently symptomatic RPF. Our patient may have benefited from colchicine 1.2 mg daily. Colchicine may be useful in treating patients with constipation from RPF. Preeclampsia (PE) is uncommon in RPF and no guidelines exist to guide the length of anticoagulation.
